# Validation of the Sleep Regularity Index in Older Adults and Associations with Cardiometabolic Risk

**DOI:** 10.1038/s41598-018-32402-5

**Published:** 2018-09-21

**Authors:** Jessica R. Lunsford-Avery, Matthew M. Engelhard, Ann Marie Navar, Scott H. Kollins

**Affiliations:** 1 Department of Psychiatry and Behavioral Sciences, Duke University Medical Center, Durham, N.C USA; 2 Duke Clinical Research Institute, Durham, NC USA

**Keywords:** Sleep Regularity, Cardiometabolic Risk, Multi-Ethnic Study Of Atherosclerosis (MESA), Delayed Sleep Timing, Irregular Sleep, Cardiovascular diseases, Sleep disorders, Risk factors

## Abstract

Sleep disturbances, including insufficient sleep duration and circadian misalignment, confer risk for cardiometabolic disease. Less is known about the association between the regularity of sleep/wake schedules and cardiometabolic risk. This study evaluated the external validity of a new metric, the Sleep Regularity Index (SRI), among older adults (n = 1978; mean age 68.7 ± 9.2), as well as relationships between the SRI and cardiometabolic risk using data from the Multi-Ethnic Study of Atherosclerosis (MESA). Results indicated that sleep irregularity was associated with delayed sleep timing, increased daytime sleep and sleepiness, and reduced light exposure, but was independent of sleep duration. Greater sleep irregularity was also correlated with 10-year risk of cardiovascular disease and greater obesity, hypertension, fasting glucose, hemoglobin A1C, and diabetes status. Finally, greater sleep irregularity was associated with increased perceived stress and depression, psychiatric factors integrally tied to cardiometabolic disease. These results suggest that the SRI is a useful measure of sleep regularity in older adults. Additionally, sleep irregularity may represent a target for early identification and prevention of cardiometabolic disease. Future studies may clarify the causal direction of these effects, mechanisms underlying links between sleep irregularity and cardiometabolic risk, and the utility of sleep interventions in reducing cardiometabolic risk.

## Introduction

Sleep is vital to supporting virtually all aspects of human health. In particular, insufficient or disturbed sleep may play an important role in the development of cardiometabolic disease^1^. Cardiometabolic diseases, including cardiovascular illness and type 2 diabetes, are leading causes of disability and death in the United States and worldwide, and are associated with significant socioeconomic costs and high rates of health care utilization^2,3^. Thus, early identification of individuals at risk for cardiometabolic illness is of high priority for the field of medicine, as implementation of preventative measures for identified individuals may have a significant, positive impact on individual patients’ health and quality of life as well as society more broadly^4^. Sleep problems represent one possible target which may aid in the identification of individuals at risk for cardiometabolic disease and provide an opportunity for preventative intervention^1^.

Sleep in humans is physiologically complex and is traditionally conceptualized as regulated by two processes – the sleep homeostasis drive (Process S), which is the accumulating pressure to sleep that increases over a period of wakefulness, and circadian rhythms (Process C), which are endogenous ~24 hour oscillations in alertness and sleep propensity that synchronize with the solar light/dark cycle^5^. There is evidence to implicate disruptions to both processes in cardiometabolic disease^1^. For example, both short and long sleep durations and poor sleep quality have been linked to increased incidence of cardiovascular disease, obesity, insulin resistance, and type 2 diabetes^6–9^. Similarly, circadian timing misalignment, in which there is a mismatch either between an individual’s internal circadian rhythm and social time or between an individual’s internal central and peripheral circadian rhythms, as well as later diurnal preference (i.e., eveningness), may also predispose individuals for cardiometabolic risk independently of the effect of sleep loss^10–12^.

An emerging aspect of sleep – the *regularity* of sleep/wake patterns – may also be important for supporting health. Specifically, individuals vary in the extent to which they are asleep and awake at the same times on a day-to-day basis, and frequent changes to the timing of sleep may contribute to circadian misalignment, as the intrinsic circadian system is slow to accommodate sleep schedule changes^13^. There is accumulating evidence – largely from studies with individuals engaged in rotating shift work – to suggest associations between irregular sleep/wake patterns and cardiometabolic disease^14–17^; however, it is unclear how generalizable these findings are to the majority of adults, who have more regular work schedules but may still display irregular sleep patterns, or older individuals, who have retired from the workforce. There is also some initial evidence from studies using self-reports, sleep diaries, and/or actigraphy linking sleep/wake irregularity to weight gain, obesity, insulin resistance, and cardiometabolic illness^18–23^. However, measures of sleep irregularity used to date may not assess extremely irregular sleep or capture rapid changes in sleep timing, which are believed to have the largest impact on regulation of the circadian system^24^.

Thus, one barrier to furthering understanding of the relationship between sleep/wake regularity and cardiometabolic illness has been the need for a measure of sleep/wake regularity that captures rapid changes in sleep timing and can also be applied to the wider adult population who have a variety of work schedules and/or are no longer in the workforce. In 2017, Phillips and colleagues proposed a novel metric for assessing this construct, the Sleep Regularity Index (SRI), which is defined as the percentage probability of a person being asleep (or awake) at any two time points 24 hours apart. The SRI differs from previous approaches in that it 1) does not require a main sleep period (and thus, is valid for use with individuals who have multiple sleep periods within a 24 hour period), and 2) is designed to capture rapid changes in sleep schedules, as it uses a day-to-day timescale^24^.

In their paper, Phillips and colleagues provided validation of the SRI in college students, and showed that the SRI is independent of sleep duration but associated with poorer subjective sleep quality and circadian misalignment (e.g., later mid-sleep time, greater “eveningness” diurnal preference). In addition, irregular sleepers, as identified by the SRI, slept less during the night and more during the day (i.e., naps) and exhibited different patterns of light exposure, including a lower amplitude of the light/dark cycle (suggesting a smaller difference in daytime and nighttime light exposure), less daytime light exposure, and more light exposure during the night, which in turn, appeared to contribute to delayed onset of melatonin secretion. Finally, greater irregularly in sleep/wake patterns was correlated with poorer academic performance, suggesting that sleep irregularity was associated with adverse consequences in their study^24^.

The Phillips study highlights the SRI as an exciting new metric for assessing sleep/wake regularity; however, its utility is furthering research in sleep and cardiometabolic risk is yet unknown. First, their investigation used a small (n = 61) young adult sample (i.e., undergraduates at Harvard University) unlikely to reflect the racial/ethnic and socioeconomic diversity of adults in the U.S. at risk for cardiometabolic disease. Second, no study to date has examined relationships between the SRI and any physical health-related outcomes. Thus, the current study builds on prior literature in several important ways. First, this study aims to examine the external validity of the SRI in a large, racially/ethnically diverse sample of older adults drawn from the Multi-Ethnic Study of Atherosclerosis (MESA)^25^. Based on the Phillips investigation, we hypothesized that sleep irregularity as measured by the SRI would be independent of sleep duration but related to delayed sleep timing in our sample. We also hypothesized that sleep irregularity would be associated with increased daytime sleepiness, greater daytime sleep, less direct light exposure, and decreased physical activity.

The second objective of the study was to examine relationships between the SRI and indices of cardiometabolic risk, including 10-year projected risk of developing cardiovascular illness, obesity, hypertension, and markers of type 2 diabetes, including fasting blood glucose and hemoglobin A1C. In addition, we investigated associations between the SRI and depressive symptoms and perceived stress, psychiatric symptoms which are found to have bidirectional associations with cardiometabolic illnesses^26–28^. Based on prior studies linking cardiometabolic disease and other measures of sleep irregularity, including rotating shift work, we hypothesized that sleep irregularity as measured by the SRI would be associated with 10 year risk of cardiometabolic disease as well as greater body mass index (BMI), hypertension, fasting blood glucose, and hemoglobin A1C. We also explored relationships between SRI and prevalent cardiovascular and metabolic diseases. Finally, we hypothesized that sleep irregularity would be associated with increased self-reported symptoms of depression and perceived stress.

## Methods

### Sample and Procedure

The current investigation is based on the Multi-Ethnic Study of Atherosclerosis (MESA), a longitudinal observational study that enrolled adults ages 45–84 free of cardiovascular disease in four racial/ethnic groups (i.e., African-American, Chinese-American, Caucasian, and Hispanic) across six regions across the United States. Participants were followed prospectively to evaluate risk factors for cardiovascular disease^25^. The baseline assessment occurred between the years of 2000 and 2002 (n = 6,814). The current analysis utilizes data collected as part of the MESA Sleep Ancillary Study^29^ (which was obtained from the National Sleep Research Resource (NSRR)^30,31^), which included a total of 2,156 individuals who completed actigraphy and self-report measures. MESA participants who reported regular use of oral devices, nocturnal oxygen, or nightly positive airway pressure were excluded from participation, and additional participants lived too far away or declined to participate in the Sleep Ancillary Study^29^. Measures included actigraphy and self-reported measures of daytime sleepiness and diurnal preference. Indices of cardiometabolic risk and psychiatric health were collected during the MESA Exam 5 assessment. Data from the Sleep Ancillary Study and MESA Exam 5 occurred between the years 2010 and 2012.

Data from the MESA Sleep Ancillary Study used in the current study are publically available from the National Sleep Research Resource repository. Data from MESA Exam 5 are available from the National Heart, Lung, and Blood Institute Biologic Specimen and Data Repository Information Coordinating Center (BioLINCC) but restrictions apply to the availability of these data, which were used under license for the current study, and so are not publicly available. Data are however available from the authors upon reasonable request and with permission of NHLBI BioLINCC. Institutional Review Board approval was obtained at each MESA study site (Wake Forest University School of Medicine, University of Minnesota, Northwestern University, Columbia University, Johns Hopkins University, and University of California -Los Angeles), and all research was performed in accordance with relevant guidelines and regulations. Written informed consent was obtained from all MESA participants.

### Measures

#### Actigraphy

Actigraphy measures of sleep/wake indices, physical activity, and light exposure were collected using the ActiWatch Spectrum (Philips Respironics), a wrist-worn device measuring physical activity and ambient light^29^. Participants wore this device for seven consecutive days while also completing a standard sleep diary^32^. Actigraphy data were aggregated in 30-second epochs and scored manually as sleep or wake using a procedure published by the NSRR and based on (a) the sleep diary, (b) activity and ambient light data from the ActiWatch device, and (c) an event marker on the ActiWatch device^30^. Participants were instructed to press the event marker when going to sleep for the evening and when awoken in the morning. To ensure adequate data collection to calculate the SRI, participants must have worn the ActiWatch device for at least five valid wear days, including at least one weekend day, to be included in the current study. Days with >2 hours of no recording (i.e. device off or not worn) were also ruled invalid and did not count toward the preceding criterion. One-hundred and eighty-one individuals were excluded based on these criteria, resulting in a final sample size of 1,978.

#### Daytime Sleepiness and Diurnal Preference

Daytime sleepiness was assessed using the self-report Epworth Sleepiness Scale (ESS), which utilizes a Likert scale (0–3) to measure excessive daytime sleepiness using eight scenarios. Scores derived from the ESS range from 0–24 with higher scores indicating greater daytime sleepiness. The ESS is a reliable, valid measure for use with adults with and without sleep disorders^33^. Diurnal preference was evaluated using a modified 5-item version of the Horne-Ostberg Morningness-Eveningness Questionnaire (MEQ), with a possible range of 4–25. Lower scores indicate greater tendency toward eveningness while higher scores reflect greater preference for morningness^34^.

#### Cardiovascular Disease and CVD Risk Factors

Cardiovascular and metabolic disease prevalence by Exam 5 was assessed as well as cardiovascular disease risk factors, including: systolic and diastolic blood pressure, low and high -density lipoprotein cholesterol (LDL-C and HDL-C) levels, triglycerides, diabetes status as determined by 2003 ADA fasting criteria^35^, metabolic syndrome by 2004 NCEP guidelines^36^, hemoglobin A1c, Body Mass Index (BMI), obesity (BMI ≥30 kg/m^2^), hypertension treatment status, coronary artery disease (prior myocardial infarction, coronary revascularization, or angina), cerebrovascular disease (prior stroke or transient ischemic attack), deep vein thrombosis pulmonary embolism (DVT/PE), coronary heart disease (CHD), and congestive heart failure (CHF). Among those free of cardiovascular disease (CVD), 10-year risk of atherosclerotic CVD (ASCVD) was calculated using the Pooled Cohorts Equations defined by the 2013 ACC/AHA guidelines^37^. This model predicts risk of a first hard ASCVD event – defined as nonfatal myocardial infarction, CHD death, or fatal or nonfatal stroke – for persons without a prior event based on demographics, blood cholesterol and blood pressure measurement, and elements of the medical history (e.g. smoking, diabetes). Ten-year ASCVD risk was also converted to age-, sex-, and race-specific percentiles^38^.

#### Psychiatric Health

The Center for Epidemiologic Studies Depression (CES-D) Scale was used as the measure of depressive symptom severity. The CES-D is a 20-item scale assessing symptoms of depression over the last week using a 0 to 3 scale. Scores range between 0 and 60 with higher scores indicating greater severity of depressive symptoms. The CES-D is associated with good reliability and validity for cross-cultural use in community-based samples^39,40^. Stress was evaluated using the 4-item Perceived Stress Scale (PSS-4), which assesses frequency with which situations in one’s life are experienced as stressful on a 5-point Likert scale. Scores range from 0 to 16, and the PSS is associated with good reliability and validity in community samples^41^.

### Calculation of Sleep Regularity Index (SRI) and other sleep indices

The Sleep Regularity Index (SRI) was originally described as “the likelihood that any two time-points (minute-by-minute) 24 hours apart were the same sleep/wake state, across all days^24^”. Our calculation uses the 30-second epochs from MESA actigraphy files but otherwise follows this description. Given *N* days of recording divided into *M* daily epochs, suppose *s*_*i,j*_ = 1 if the participant was sleeping on day *i* in epoch *j*, and *s*_*i,j*_ = 0 if they were awake. Then, the SRI was calculated as equation (1):

1 $$-100+\frac{200}{M(N-1)}\sum _{j=1}^{M}\sum _{i=1}^{N-1}\delta ({s}_{i,j},{s}_{i+1,j})$$

where $\delta ({s}_{i,j},{s}_{i+1,j})=1\,{\rm{if}}\,{s}_{i,j}={s}_{i+1,j}$ and 0 otherwise.

Regarding other sleep-related variables, sleep duration (total sleep time; TST) was calculated as average daily (24-hour) sleep time in minutes in equation (2), i.e.

2 $${\sum }_{i,j}{s}_{i,j}\times 0.5\,{\rm{minutes}}/N\,{\rm{days}}.$$

Sleep midpoint, our index of sleep timing, was calculated as a mean of circular quantities (appropriate for time of day) using the following equation (3), where *t*_*j*_ denotes time of day in minutes at epoch *j*:

3 $$\frac{1440}{2\pi }\arctan 2(\sum _{j=1}^{M}\sum _{i=1}^{N}{s}_{i,j}\,\sin \,\frac{2\pi {t}_{i}}{1440}+\sum _{j=1}^{M}\sum _{i=1}^{N}{s}_{i,j}\,\cos \,\frac{2\pi {t}_{i}}{1440})$$

Finally, average daily activity was calculated as the sum of all activity counts divided by *N* days.

### Statistical Analyses

Analyses were conducted in Python 3.5 with SciPy^42^ and Matplotlib^43^. Associations between sleep indices (SRI, TST, midpoint) and numeric clinical variables – including age, BMI and cardiometabolic risk, and measures of psychiatric health – have been quantified as partial linear correlations with age, sex, and race/ethnicity as control variables. Partial correlations were calculated as the Pearson correlation between residuals following multiple regression of both variables of interest on the control variables. Relationships between pairs of actigraphy-derived values (sleep indices and physical activity) have been reported as raw Pearson correlations to illustrate direct, mathematical relationships between these calculated quantities. Pearson correlation was also used to quantify associations between sleep indices and the aforementioned age, sex, and race-stratified risk percentiles, as controlling for these demographics a second time via partial correlation would not be appropriate. Second-order (quadratic) relationships between sleep indices and cardiometabolic variables were assessed by applying quadratic regression to residuals obtained after multiple regression of both variables of interest on the control variables. Examining second-order relationships was particularly important for sleep duration and cardiometabolic risk, as both long and short sleep durations have been tied to increased mortality from cardiovascular disease^6^.

Sleep indices have also been compared between groups defined by categorical clinical variables (e.g. hypertension, cardiometabolic disease). These comparisons were made by nonparametric Mann-Whitney U test unless normality was established by Kolmogorov-Smirnov test, in which case a t-test was used. Numeric clinical variables have also been compared between irregular sleepers, defined as participants with SRI values in the first quintile, and regular sleepers, defined as participants with SRI values in the fifth quintile, as has been done previously^24^. As before, comparisons were assessed by Mann-Whitney U test unless variables were normally distributed. Effect sizes have been quantified using Cohen’s *d* metric^44^. Relationships between pairs of categorical variables (e.g. educational status vs SRI group) have been quantified by chi-square test. Note that the central tendency of non-normal and ordinal variables (including SRI) has been assessed using the median rather than the mean.

Due to the large number of comparisons in this analysis (approximately 400) as well as the substantial statistical power of this dataset, relationships are considered statistically significant only when p < 10^−4^ (i.e. Bonferroni correction). Trends not satisfying this criterion have been identified in the analyses of prevalent disease due to the comparatively modest number of participants in each disease category.

## Results

### External Validation of the SRI in the MESA sample

#### Associations between SRI and demographic variables

SRI values ranged from 6.2 to 97.0 (mean 71.6 ± 14.5), and were negatively skewed (median = 74.7) and non-normal (p < 10^−4^). SRI values of 60.8 and 84.0 defined the 20^th^ and 80^th^ percentiles, respectively, and were used to group participants as irregular (SRI < 60.8) and regular (SRI ≥ 84.0). See Supplementary Fig. S1.

Table 1 describes demographic characteristics for the overall sample as well as group differences in demographic variables between irregular and regular sleepers. Regarding demographics, SRI trended toward being higher in women (mean 72.4 ± 13.9) than in men (mean 70.7 ± 15.2) (p = 0.021) and decreasing with age (r = −0.058, p = 0.001), but neither was statistically significant after Bonferroni correction. Similarly, while SRI trended toward being higher among individuals in the workforce (including full- and part-time employment) than those not currently working, this difference did not reach statistical significance after correcting for multiple comparisons (p = 0.004). SRI did not differ by educational status (p = 0.69).

**Table 1:** Demographic characteristics among all participants as well as irregular sleepers (individuals in the first quintile based on SRI) and regular sleepers (fifth SRI quintile).

	All (n = 1978)	Irregular (n = 396)	Regular (n = 396)	p-value
Mean (SD) [Range]				
Age	68.7 (9.2) [54–93]	69.6 (10.1) [54–93]	68.0 (8.8) [54–90]	0.001
N (%)				
Sex (Female)	1063 (54%)	188 (47%)	221 (56%)	0.022
Racial/Ethnic Group				< 10^−4^
*Caucasian*	762 (39%)	92 (23%)	209 (53%)	
*Chinese-American*	205 (10%)	36 (9%)	36 (9%)	
*African-American*	561 (28%)	171 (43%)	67 (17%)	
*Hispanic*	449 (23%)	96 (24%)	84 (21%)	
Highest Education Completed				0.69
*No Degree*	274 (14%)	58 (15%)	49 (12%)	
*High School Degree/GED*	325 (16%)	78 (20%)	69 (17%)	
*Some College, No Degree*	321 (16%)	61 (15%)	57 (14%)	
*College Degree (2 or 4 year)*	658 (33%)	129 (33%)	137 (35%)	
*Graduate or Professional School*	396 (20%)	68 (17%)	83 (21%)	
Employment Status				0.004
*Homemaker*	178 (9%)	37 (9%)	35 (9%)	
*Currently Working*	1042 (53%)	187 (47%)	205 (52%)	
*Not Currently Working*	106 (5%)	37 (9%)	16 (4%)	
*Retired*	646 (33%)	134 (34%)	138 (35%)	

However, differences between the four racial/ethnic groups were statistically significant: SRI was lower among African-American participants compared to all other groups (p < 10^−5^) and lower among Hispanic participants compared to Caucasian participants (p < 10^−5^), with a trend observed between Chinese-American and Caucasian participants (p = 0.0008), suggesting greater sleep irregularity among minority individuals compared to Caucasians.

#### Relationships between the SRI and TST, sleep timing, and physical activity

**Figure 1:**
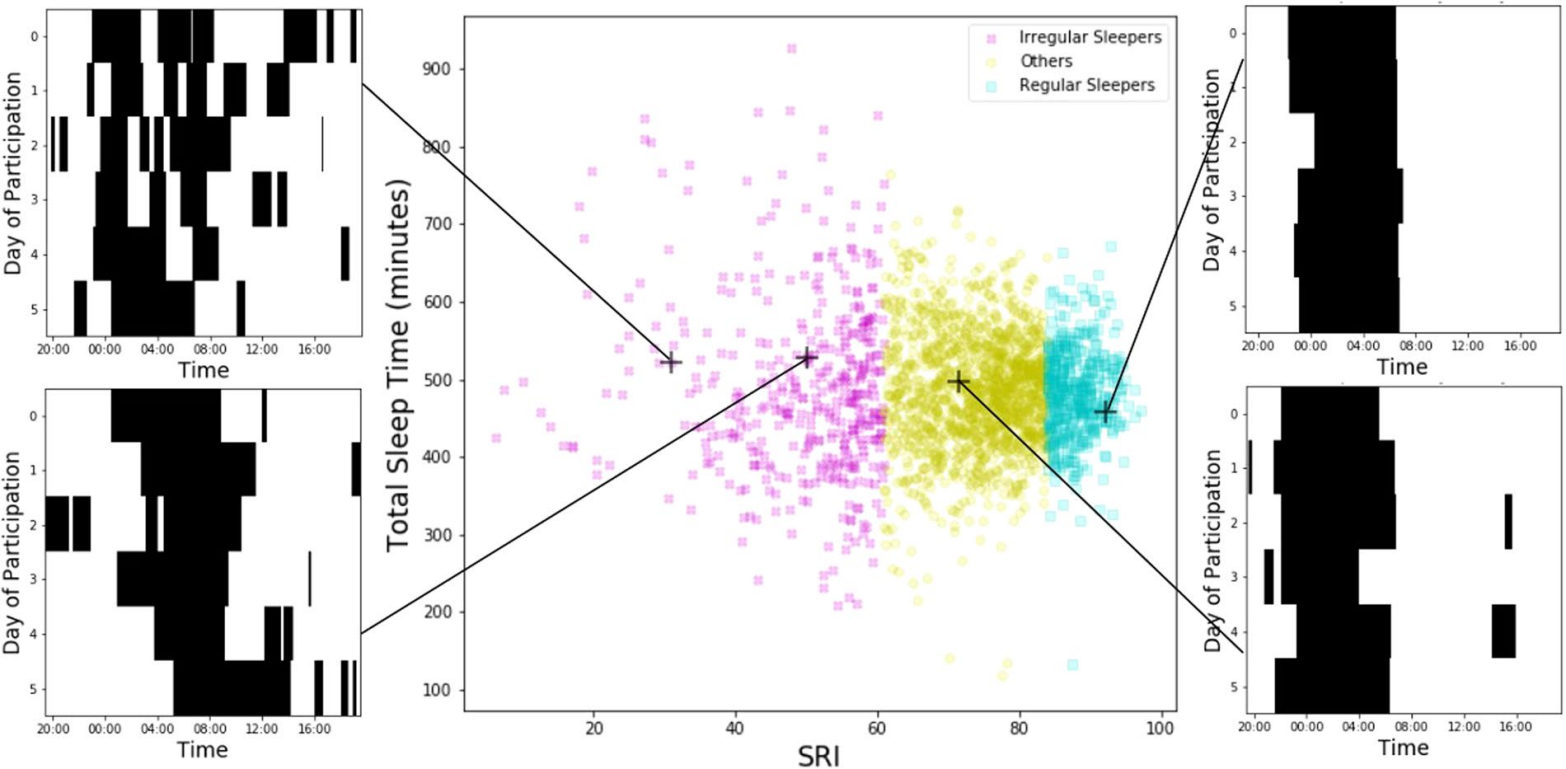
Panel A: Relationship between SRI and TST and illustration of the effect of daily sleep/wake patterns on SRI using four example subjects. Panels B and C: Daily schedules for two irregular sleepers. Panels D and E: Daily schedules for a regular sleeper and a participant not in either group. Results from the current analysis are shown, but the design of this figure is based on a similar figure created by Phillips *et al*.^19^.

SRI was not significantly correlated with TST (r = −0.051, p = 0.022, Fig. 1), but was negatively correlated with sleep midpoint (r = −0.227, p < 10^−23^; Supplementary Fig. S2), meaning that those with less regular sleep tended to sleep later in the day. SRI was also positively correlated with average daily activity (r = 0.192, p < 10^−17^; Supplementary Fig. S3), showing that those with more regular sleep tended to be more active. Group comparisons indicated that irregular sleepers had a later sleep midpoint compared to regular sleepers, but similar TST. Irregular sleepers were also less physically active compared to regular sleepers. See Table 2.

**Table 2:** Group differences in sleep-related variables among irregular and regular sleepers.

	Irregular	Regular	p-value	Cohen’s d
	Median (IQR)			
Actigraphy Measures				
Total Sleep Time (minutes)	477.2 (142.1)	480.6 (74.6)	0.604	0.089
Sleep Midpoint (clock time)	03:36 am (2:17)	02:59 am (1:14)	<10^−9^	0.426
Physical Activity (counts in thousands)	184.3 (108.8)	229.3 (97.1)	<10^−13^	0.517
Sleep in Clock Day (minutes)	74.5 (76.1)	8.0 (18.5)	<10^−96^	1.551
Sleep in Clock Night (minutes)	406.9 (122.1)	467.3 (65.8)	<10^−29^	0.882
Daily Light Exposure (minutes >250 lux)	86.0 (109.5)	134.4 (141.7)	<10^−11^	0.488
Self-Report Measures				
ESS	6 (6)	4 (5)	<10^−15^	0.595
MEQ	17 (5)	18 (5)	<10^−4^	0.315

#### Relationships between the SRI and daytime sleepiness and diurnal preference

SRI was negatively and significantly correlated with daytime sleepiness (pr = −0.200, p < 10^−18^) and the group difference in median ESS score between irregular sleepers and regular sleepers was statistically significant. SRI was also significantly correlated with diurnal preference (pr = 0.150, p < 10^−10^), with more irregular sleep patterns associated with greater preference for eveningness. The group difference in median MEQ score among irregular sleepers compared to regular sleepers was statistically significant. See Table 2.

#### Group differences in sleep timing and light exposure

**Figure 2:**
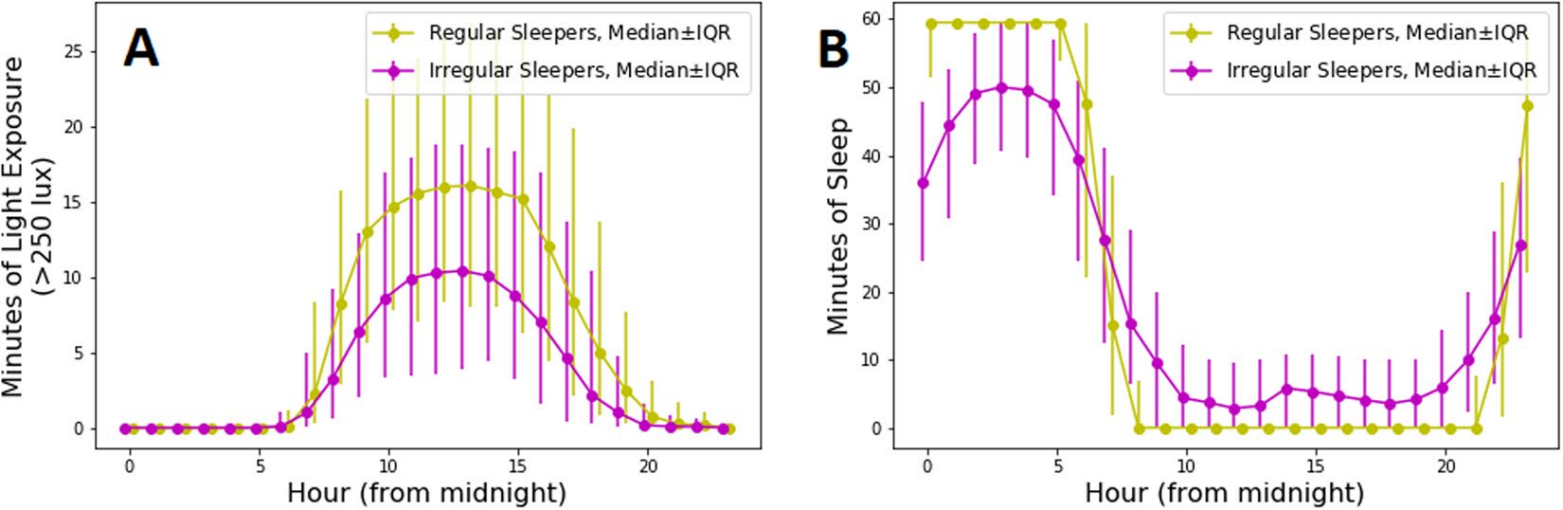
Sleep/wake and light/dark patterns for irregular and regular sleepers. Panel A: Direct light exposure by hour. Panel B: sleep by hour.

Irregular sleepers were delayed in their sleep patterns, with less sleep during nighttime hours (clock night = 22:00 pm–10:00 am) and more sleep during daytime hours (clock day = 10:00 am–22:00 pm). Specifically, on average, irregular sleepers slept for 66.5 minutes more during the clock day and 60.4 minutes less during the clock night compared to regular sleepers. In addition, irregular sleepers tend to be exposed to fewer minutes of light (>250 lux). On average, irregular sleepers were exposed to 48.4 fewer minutes of light per day than regular sleepers. See Fig. 2 and Table 2.

### Associations between sleep regularity and cardiometabolic profile

#### Relationships between sleep regularity and cardiovascular risk

**Figure 3:**
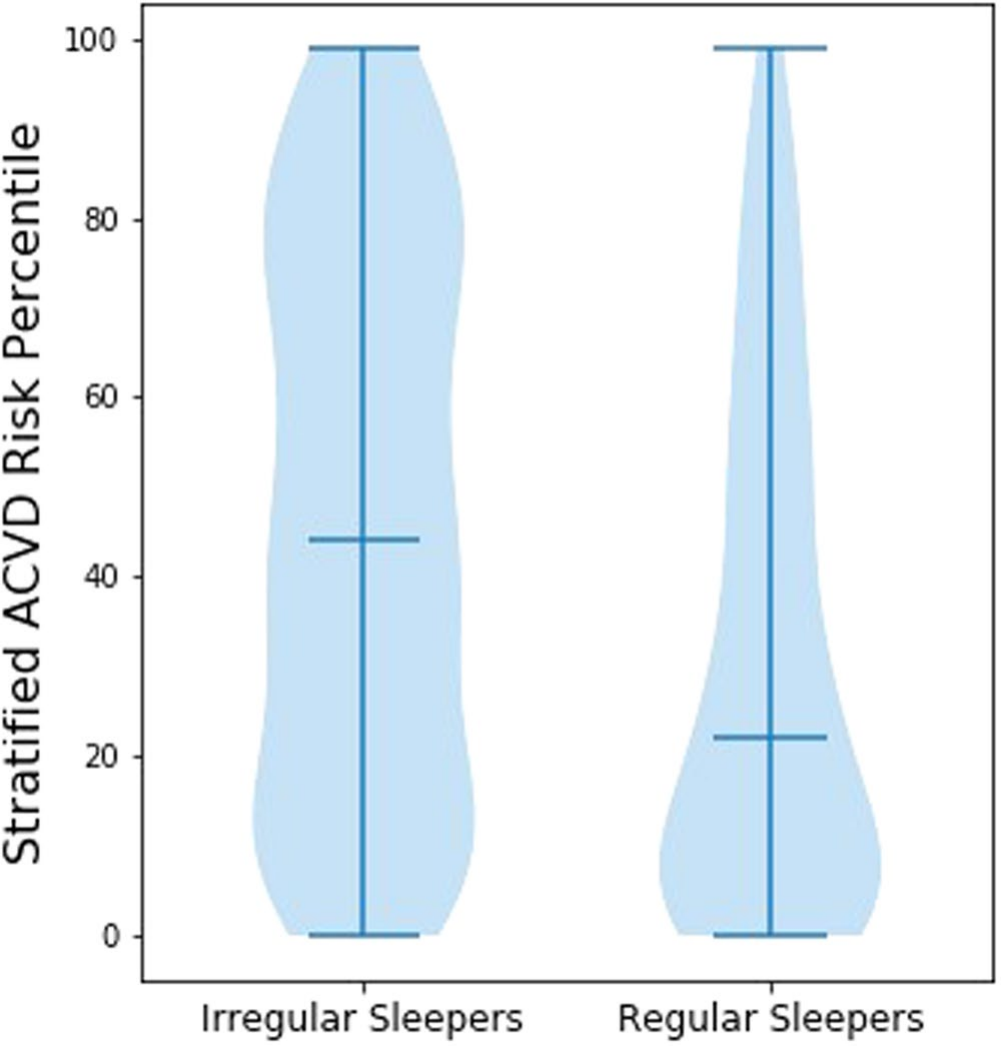
Age, sex, and race-stratified ASCVD risk percentile by SRI group among participants without prevalent ASCVD.

Ten-year risk of atherosclerotic cardiovascular disease (ASCVD) was significantly correlated with SRI (pr = −0.133, p < 10^−8^) among participants without prevalent ASCVD. Further, converting these risk scores to relative risk stratified by race, sex, and age revealed an even stronger association with SRI (r = −0.164, p < 10^−8^). ASCVD risk was not significantly associated with sleep midpoint (pr = 0.016, p = 0.486) or TST (pr = 0.032, p = 0.155). There was a statistically significant second-order (quadratic) relationship between risk and TST (p < 10^−4^), but the conversion to relative risk eliminated this relationship (p = 0.296). ASCVD risk and cardiometabolic variables are compared between irregular sleepers and regular sleepers in Fig. 3 and Table 3.

**Table 3:** Group differences in ASCVD risk and cardiometabolic variables among irregular and regular sleepers.

	Irregular	Regular	p-value	Cohen’s d
	Median (IQR)			
10-year ASCVD Risk	0.162 (0.236)	0.109 (0.158)	<10^−7^	0.379
Stratified ASCVD Risk Percentile	46.0 (58.5)	22.0 (46.0)	<10^−9^	0.553
Seated Systolic Blood Pressure (mmHg)	121.5 (26.0)	117.0 (24.5)	0.0004	0.283
Seated Diastolic Blood Pressure (mmHg)	68.5 (14.9)	66.5 (12.0)	0.0061	0.225
BMI (kg/m^2^)	29.7 (7.7)	27.0 (7.2)	<10^−7^	0.391
Serum Hemoglobin A1C	6.0 (0.9)	5.7 (0.5)	<10^−10^	0.449
Fasting Blood Glucose (mg/dl)	99.0 (23.0)	94.0 (14.0)	<10^−6^	0.356

Lower SRI was also associated with higher BMI (r = −0.139, p < 10^−9^, pr = −0.124, p < 10^−7^), higher hemoglobin A1C (pr = −0.138, p < 10^−9^), and higher fasting blood glucose (pr = −0.105, p < 10^−5^). Sleep midpoint and TST were not significantly associated with BMI (pr = 0.069, p = 0.002; and pr = −0.005, p = 0.808, respectively), hemoglobin A1C (pr = 0.060, p = 0.009; pr = 0.028, p = 0.219, respectively), or fasting blood glucose (pr = 0.054, p = 0.016; pr = 0.041, p = 0.067, respectively). Similarly, in terms of group comparisons, irregular sleepers displayed greater BMI, hemoglobin AIC, and fasting blood glucose than regular sleepers. See Table 3.

#### Associations between SRI and Cardiometabolic Disease

SRI was significantly lower among participants with metabolic syndrome (median = 71.7) compared to those without (median = 76.2) (p < 10^−8^); lower among those with hypertension (median 72.9) versus those without (median 76.8) (p < 10^−7^); lower among those with diabetes mellitus (median = 68.5) versus those without (median = 75.8) (p < 10^−12^); and lower among those with obesity (BMI ≥30) (median = 72.6) versus those without (median = 75.8, p < 10^−6^). TST also differed by hypertension status (median 487 versus 475 minutes, p < 10^−4^), with those with hypertension getting more sleep on average, and sleep midpoint differed by obesity status (median 03:05 am versus 03:18 am, p < 10^−4^), with those with BMI ≥30 having a later midpoint. Otherwise TST and sleep midpoint did not differ between the aforementioned groups (p > 10^−4^).

**Figure 4:**
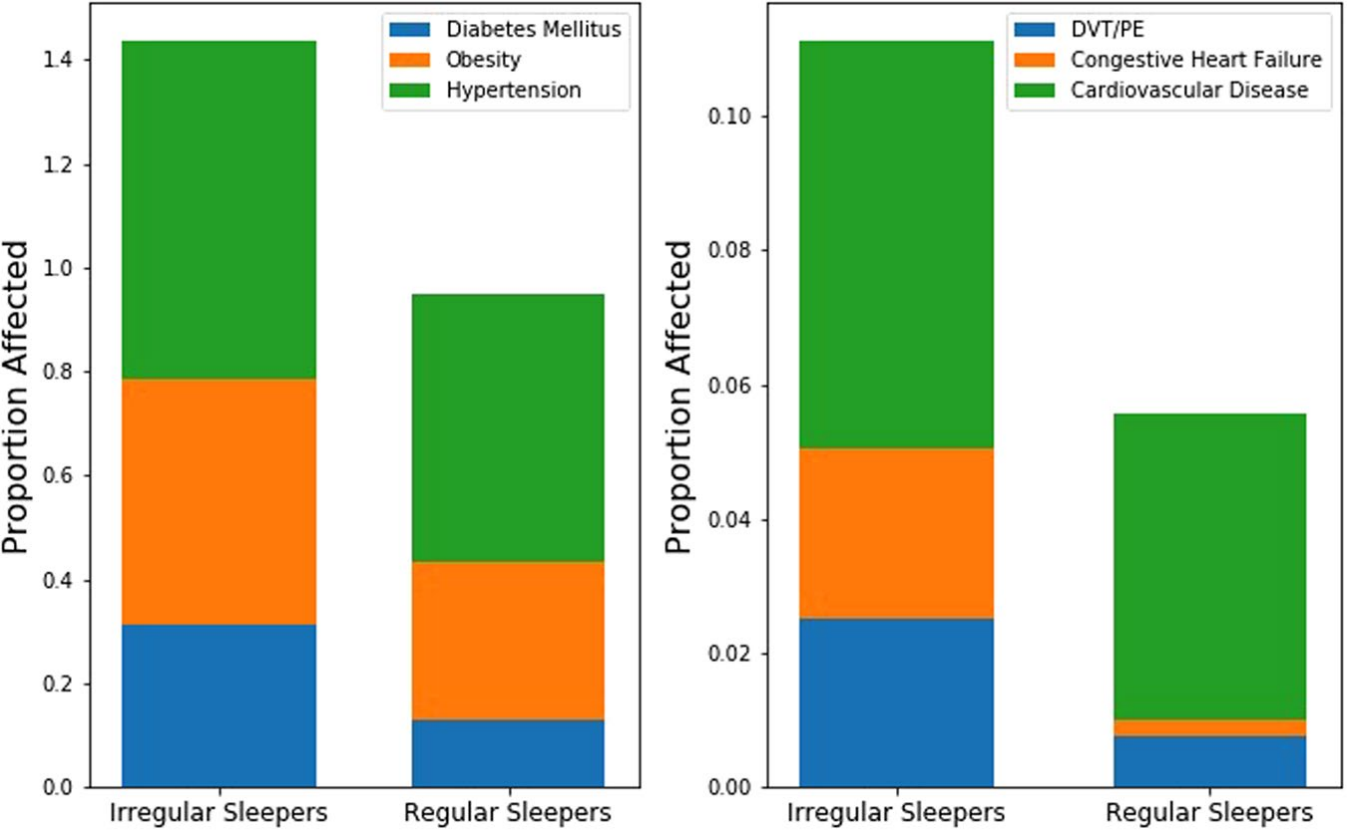
Proportion of participants in each SRI group with diabetes mellitus, obesity (BMI ≥ 30 kg/m^2^), and hypertension (Panel A) as well as cardiovascular disease (includes coronary heart disease, stroke, stroke death, other atherosclerotic death, and other cardiovascular disease death), congestive heart failure, and deep vein thrombosis/pulmonary embolism (Panel B). The downward trends show that subjects with less regular sleep (lower SRI) were more likely to have experienced these events.

Trends were observed between participants with/without prevalent CHF (29 participants; median SRI 67.3 and 74.3, respectively; p = 0.005), documented DVT/PE (29 participants; median SRI 66.0 and 74.7, respectively; p = 0.015), and prevalent CHD (77 participants; median SRI 69.4 and 74.9, respectively; p = 0.017); but not ASCVD (93 participants; median SRI 71.2 and 74.8, respectively; p = 0.198). In contrast, a trend in TST was observed only between subjects with/without prevalent ASCVD (93 participants; median TST 8:21 and 8:01, respectively; p = 0.045). No other trends in TST or sleep midpoint were observed between these groups (p > 0.05). To compliment these analyses, Fig. 4 compares rates of diabetes, obesity, hypertension, DVT/PE, CHF, and ASCVD between irregular and regular sleepers. Rates of obesity and diabetes mellitus differ between irregular and regular sleepers (p < 10^−4^), and trends in rates of hypertension and CHF were also observed (p < 0.05).

#### Associations between SRI and psychiatric health

**Figure 5:**
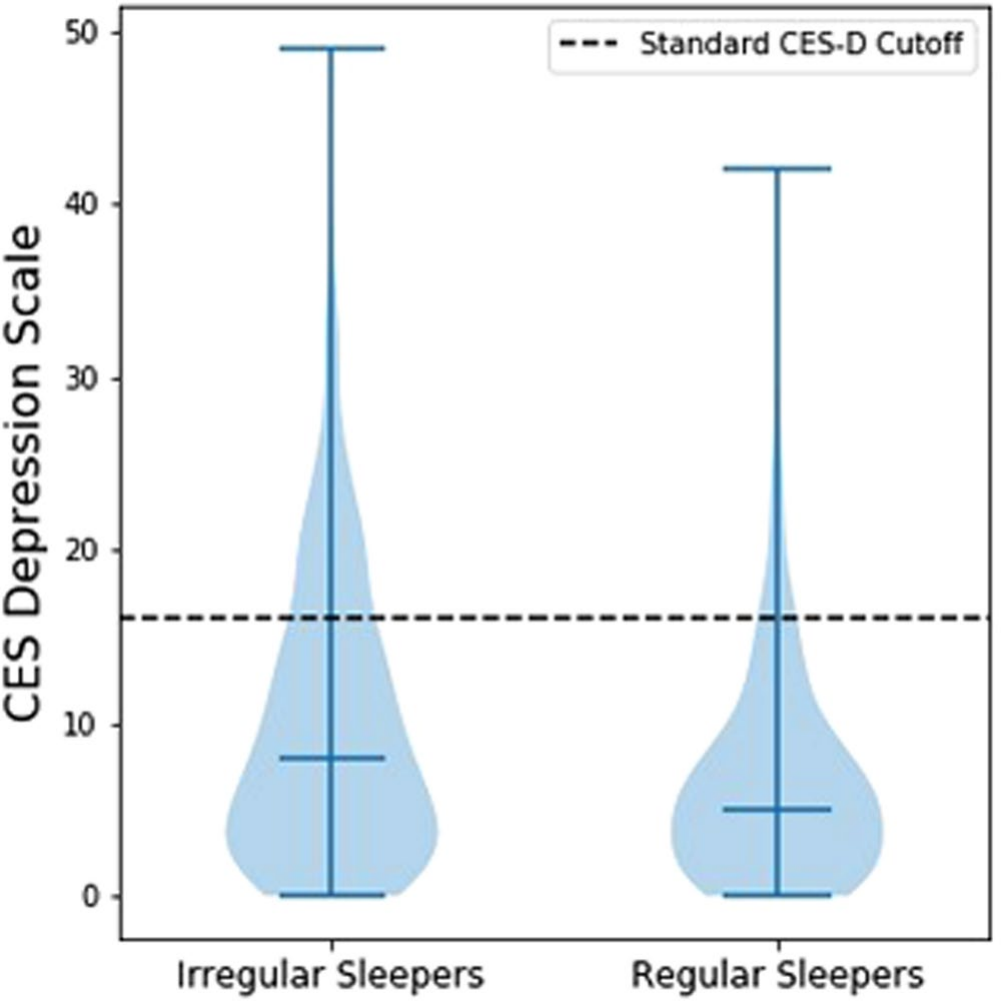
Group differences in depression among irregular and regular sleepers.

Controlling for age, sex, and race, lower SRI was significantly associated with increased self-reported depressive symptom severity (pr = −0.132, p < 10^−8^) and perceived stress (pr = −0.100, p < 10^−4^). In contrast, TST, sleep midpoint, and total daily activity were not significantly associated with either outcome (p > 10^−4^). Regarding group comparisons, irregular sleepers tended to have higher scores on the CES-D compared to regular sleepers (p < 10^−6^). SRI group (irregular/regular) was significantly associated with CES-D score ≥ 16, the standard cutoff for predicted depression (p < 10^−5^). Indeed, irregular sleepers had 2.53 increased odds of exceeding the CES-D cutoff (95% CI 1.67–3.84) compared to regular sleepers. See Fig. 5.

## Discussion

This study is the first to examine 1) the external validity of a novel metric of sleep regularity, the SRI, in racially/ethnically-diverse older adults and 2) associations between the SRI and physical health outcomes, specifically cardiometabolic risk. Regarding the first aim, results suggested that sleep irregularity was associated with delayed sleep timing and eveningness preference but was independent of sleep duration. In addition, irregular sleep/wake patterns were related to reduced physical activity, increased daytime sleep and sleepiness, and reduced light exposure. These findings are highly consistent with results reported by Phillips and colleagues in their college student sample, supporting the external validity of the SRI with older adults^24^. Notably, the SRI differed significantly by racial/ethnic identity in the current study, with Caucasian Americans displaying the highest sleep regularity and African Americans exhibiting the greatest sleep irregularity. This pattern is similar to a prior report, which has also shown an association between sleep irregularity and minority race using another metric of sleep regularity (i.e., variability in sleep duration)^45^.

In regard to the second objective, results revealed significant relationships between sleep irregularity and indices of cardiometabolic risk, including obesity and higher levels of fasting blood glucose and hemoglobin AIC. In addition, sleep irregularity was associated with higher projected risk of developing atherosclerotic cardiovascular disease over the next decade, and individuals with hypertension and/or metabolic disease displayed higher sleep irregularity than those without. Greater sleep irregularity was further correlated with increased depression severity and perceived stress, aspects of psychiatric health known to have bidirectional relationships with cardiometabolic disease. Finally, trends were observed between greater sleep irregularity and prevalent congestive heart failure, prevalent coronary heart disease, and documented deep vein thrombosis and/or pulmonary embolism.

These results support and extend prior literature linking irregular sleep/wake patterns to increased cardiometabolic risk in adults. First, the current study suggests that relationships between sleep irregularity and cardiometabolic risk extend beyond individuals with rotating shift work schedules to the broader population who may have more stable work patterns and/or have left the work force. Second, this investigation suggests that the SRI, a novel measure designed to detect rapid changes in sleep schedules as well as adequately characterize the sleep/wake patterns of extremely irregular sleepers, is sensitive to capturing sleep/wake irregularity that may confer risk for developing cardiometabolic disease. This finding dovetails with prior studies linking various indices of sleep irregularity – such as social jetlag, bedtime variability, standard deviation of bedtime, standard deviation of mid-sleep time, and standard deviation of sleep duration – to indices of cardiometabolic health^18–23^. Although our findings linking the SRI and cardiometabolic risk support the external validity of the measure, the relative sensitivity of the SRI compared to other measures of sleep irregularity in predicting health outcomes remains an open question and should be the focus of future study.

Interestingly, more frequently studied indices of sleep/wake functioning, including sleep duration and sleep timing, were less often significantly related to cardiometabolic outcomes than sleep regularity in the current study. Increased sleep duration was observed in individuals with hypertension compared to those without, and obese individuals evidenced a later sleep midpoint than non-obese individuals. There were also trends toward relationships between insufficient sleep duration and/or delayed sleep timing and several other indices of cardiometabolic risk in expected directions. In general, however, associations between cardiometabolic risk and sleep duration/timing were less robust than associations with sleep regularity. This pattern of results highlights the importance of considering regularity in addition to sleep duration, quality, timing, and diurnal preference when assessing potential links between sleep and cardiometabolic illness, and indeed, future work should investigate the relative contributions of each of these aspects of sleep/wake function – and possible interactions among them – to cardiometabolic risk.

Our findings have important research and clinical implications. First, this study highlights the utility of the SRI for measuring sleep regularity in adults across the lifespan and from a variety of backgrounds. Second, results highlight sleep irregularity as a possible risk factor for cardiometabolic disease. Although the direction of the relationship is yet unknown (i.e., sleep irregularity may result in cardiometabolic risk or vice versa, or the association may be bidirectional), there is some evidence from animal and human studies to suggest that sleep/wake disturbances may directly confer risk for cardiometabolic illness through several mechanisms, including interference with energy metabolism, glucose metabolism, and timing of food intake and their resulting impact on body composition^1^. In addition, sleep irregularity was associated with increased stress and depression in this study, which may suggest additional mechanisms through which sleep irregularity may contribute to cardiometabolic risk, as stress and depression are also associated with 10-year risk of cardiovascular disease and appear to play causal roles in illness onset^46,47^.

Thus, sleep irregularity may represent a potential target for early identification of individuals at risk for cardiometabolic disease and provide opportunities for prevention and intervention efforts, and indeed, recent technological innovations may facilitate detection and treatment opportunities in the broader population. For example, advances in sleep/wake pattern detection using smartphones are underway and may aid in detection of sleep irregularity^48^. In addition, brief behavioral treatments such as cognitive-behavioral therapy for insomnia (CBT-I) enhance sleep/wake regularity by specifying consistent bed- and rise-times and can be effaciously delivered via the internet, increasing access to the public^49,50^. Future research is necessary to elucidate the utility of emerging technologies in identifying and ameliorating irregular sleep/wake patterns conferring risk for cardiometabolic illness.

This study is subject to several limitations. First, analyses were largely cross-sectional. Thus, the current study does not shed light on the direction of causation in the associations between sleep irregularity and cardiometabolic risk. Second, although this investigation utilized a large sample, analyses investigating associations between sleep irregularity and prevalent cardiovascular and metabolic diseases were underpowered due to the low base rate of these diseases experienced by participants. Future studies utilizing a longitudinal design assessing both sleep irregularity and indices of cardiometabolic risk at each time point may clarify both causal mechanisms as well as relationships between sleep irregularity and cardiometabolic disease.

Third, it is notable that we were unable to exclude shift workers from our analyses, as work schedule information was not collected as part of MESA. Although this represents a potential confound, as shift workers are at elevated risk for cardiometabolic outcomes^14–17^, we believe this to be unlikely in the current study given the large proportion of our sample not in the workforce (47%), the relatively low rate of shift work among those with fulltime employment in the U.S. population (15%)^51^, and our finding in the present study that employed individuals in general displayed relatively *better* sleep regularity compared to those not in the workforce. Nonetheless, individuals’ engagement in shift work should be considered in future studies assessing sleep regularity and cardiometabolic outcomes in adults. Finally, it is notable that in the original investigation of the SRI conducted by Phillips and colleagues, SRI was shown to be related to delayed circadian phase, as indicated by timing of endogenous melatonin rhythms, among college students, which may be due to insufficient daytime light exposure among irregular sleepers^24^. Future studies should assess whether an analogous relationship exists between the SRI and circadian phase among older, racially/ethnically diverse adults using dim-light melatonin onset (DLMO) and related methods.

## Electronic supplementary material

Supplementary Information

## References

[1] McHill AW, Wright KP (2017). Role of sleep and circadian disruption on energy expenditure and in metabolic predisposition to human obesity and metabolic disease. Obes Rev.

[2] American Diabetes, A. Economic Costs of Diabetes in the U.S. in 2017. *Diabetes Care*, 10.2337/dci18-0007 (2018).

[3] Benjamin EJ (2018). Heart Disease and Stroke Statistics-2018 Update: A Report From the American Heart Association. Circulation.

[4] Kazi DS (2018). Building the Economic Case for Investment in Cardiovascular Prevention. J Am Coll Cardiol.

[5] Borbely AA, Daan S, Wirz-Justice A, Deboer T (2016). The two-process model of sleep regulation: a reappraisal. J Sleep Res.

[6] Krittanawong C (2017). Association between Short and Long Sleep Duration and Cardiovascular Outcomes? A Systematic Review and Meta-Analysis. Journal of the American College of Cardiology.

[7] Shan Z (2015). Sleep duration and risk of type 2 diabetes: a meta-analysis of prospective studies. Diabetes Care.

[8] Wu YL, Zhai L, Zhang DF (2014). Sleep duration and obesity among adults: a meta-analysis of prospective studies. Sleep Med.

[9] Hoevenaar-Blom MP, Spijkerman AM, Kromhout D, van den Berg JF, Verschuren WM (2011). Sleep duration and sleep quality in relation to 12-year cardiovascular disease incidence: the MORGEN study. Sleep.

[10] Morris CJ, Purvis TE, Hu K, Scheer FAJL (2016). Circadian misalignment increases cardiovascular disease risk factors in humans. P Natl Acad Sci USA.

[11] Leproult R, Holmback U (2014). & Van Canter, E. Circadian Misalignment Augments Markers of Insulin Resistance and Inflammation, Independently of Sleep Loss. Diabetes.

[12] Knutson, K. L. & von Schantz, M. Associations between chronotype, morbidity and mortality in the UK Biobank cohort. *Chronobiol Int*, 1–9, 10.1080/07420528.2018.1454458 (2018). 10.1080/07420528.2018.1454458 PMC6119081 29642757

[13] Wittmann M, Dinich J, Merrow M, Roenneberg T (2006). Social jetlag: Misalignment of biological and social time. Chronobiology International.

[14] Barbadoro Pamela, Santarelli Lory, Croce Nicola, Bracci Massimo, Vincitorio Daniela, Prospero Emilia, Minelli Andrea (2013). Rotating Shift-Work as an Independent Risk Factor for Overweight Italian Workers: A Cross-Sectional Study. PLoS ONE.

[15] Lieu SJ, Curhan GC, Schernhammer ES, Forman JP (2012). Rotating night shift work and disparate hypertension risk in African-Americans. J Hypertens.

[16] Pan An, Schernhammer Eva S., Sun Qi, Hu Frank B. (2011). Rotating Night Shift Work and Risk of Type 2 Diabetes: Two Prospective Cohort Studies in Women. PLoS Medicine.

[17] Vetter C (2016). Association Between Rotating Night Shift Work and Risk of Coronary Heart Disease Among Women. Jama-J Am Med Assoc.

[18] Sohail S, Yu L, Bennett DA, Buchman AS, Lim AS (2015). Irregular 24-hour activity rhythms and the metabolic syndrome in older adults. Chronobiol Int.

[19] Parsons MJ (2015). Social jetlag, obesity and metabolic disorder: investigation in a cohort study. Int J Obes (Lond).

[20] Taylor BJ (2016). Bedtime Variability and Metabolic Health in Midlife Women: The SWAN Sleep Study. Sleep.

[21] Bailey BW (2014). Objectively measured sleep patterns in young adult women and the relationship to adiposity. Am J Health Promot.

[22] Kobayashi D (2013). High sleep duration variability is an independent risk factor for weight gain. Sleep Breath.

[23] Roane BM (2015). What Role Does Sleep Play in Weight Gain in the First Semester of University?. Behav Sleep Med.

[24] Phillips, A. J. K. *et al*. Irregular sleep/wake patterns are associated with poorer academic performance and delayed circadian and sleep/wake timing. *Sci Rep-Uk* **7**, ARTN 3216 10.1038/s41598-017-03171-4 (2017). 10.1038/s41598-017-03171-4 PMC5468315 28607474

[25] Bild DE (2002). Multi-Ethnic Study of Atherosclerosis: objectives and design. Am J Epidemiol.

[26] Golden SH (2008). Examining a bidirectional association between depressive symptoms and diabetes. Jama-J Am Med Assoc.

[27] Whooley MA, Wong JM (2013). Depression and Cardiovascular Disorders. Annu Rev Clin Psycho.

[28] Rosmond R (2005). Role of stress in the pathogenesis of the metabolic syndrome. Psychoneuroendocrino.

[29] Chen X (2015). Racial/Ethnic Differences in Sleep Disturbances: The Multi-Ethnic Study of Atherosclerosis (MESA). Sleep.

[30] Dean DA (2016). Scaling Up Scientific Discovery in Sleep Medicine: The National Sleep Research Resource. Sleep.

[31] Zhang Guo-Qiang, Cui Licong, Mueller Remo, Tao Shiqiang, Kim Matthew, Rueschman Michael, Mariani Sara, Mobley Daniel, Redline Susan (2018). The National Sleep Research Resource: towards a sleep data commons. Journal of the American Medical Informatics Association.

[32] Morgenthaler TI (2007). Practice parameters for the clinical evaluation and treatment of circadian rhythm sleep disorders. An American Academy of Sleep Medicine report. Sleep.

[33] Johns MW (1992). Reliability and factor analysis of the Epworth. Sleepiness Scale. Sleep.

[34] Horne JA, Ostberg O (1976). A self-assessment questionnaire to determine morningness-eveningness in human circadian rhythms. Int J Chronobiol.

[35] Genuth S (2003). Follow-up report on the diagnosis of diabetes mellitus. Diabetes Care.

[36] Grundy SM (2004). Definition of metabolic syndrome - Report of the National Heart, Lung, and Blood Institute/American Heart Association Conference on Scientific Issues Related to Definition. Circulation.

[37] Goff DC (2014). 2013 ACC/AHA guideline on the assessment of cardiovascular risk: a report of the American College of Cardiology/American Heart Association Task Force on Practice Guidelines. J Am Coll Cardiol.

[38] Navar AM, Pencina MJ, Mulder H, Elias P, Peterson ED (2018). Improving patient risk communication: Translating cardiovascular risk into standardized risk percentiles. Am Heart J.

[39] Radloff LS (1977). The CES-D scale: A self-report depression scale for research in the general population. Applied Psychological Measurement.

[40] Roberts RE (1980). Reliability of the CES-D Scale in different ethnic contexts. Psychiatry Res.

[41] Cohen S, Kamarck T, Mermelstein R (1983). A global measure of perceived stress. J Health Soc Behav.

[42] Oliphant TESP (2007). Open source scientific tools for Python. Computing in Science and Engineering.

[43] Hunter JD (2007). Matplotlib: A 2D graphics environment. Computing in science & engineering.

[44] Cohen, J. *Statistical power analysis for the behavioral sciences*. 2nd edn, (L. Erlbaum Associates, 1988).

[45] Patel SR (2014). The association between sleep patterns and obesity in older adults. Int J Obesity.

[46] Kyrou I (2017). Association of depression and anxiety status with 10-year cardiovascular disease incidence among apparently healthy Greek adults: The ATTICA Study. *Eur*. J Prev Cardiol.

[47] Kivimaki, M. & Kawachi, I. Work Stress as a Risk Factor for Cardiovascular Disease. *Curr Cardiol Rep* **17**, ARTN 74 10.1007/s11886-015-0630-8 (2015). 10.1007/s11886-015-0630-8 PMC4523692 26238744

[48] Abdullah, S., Murnane, E. L., Matthews, M. & Choudhury, T. In *Mobile Health* (eds Rehg, J. M., Murphy, S. A. & Kumar, S.) 35–58 (Springer, 2017).

[49] Zachariae R, Lyby MS, Ritterband LM, O’Toole MS (2016). Efficacy of internet-delivered cognitive-behavioral therapy for insomnia - A systematic review and meta-analysis of randomized controlled trials. Sleep Med Rev.

[50] Seyffert M (2016). Internet-Delivered Cognitive Behavioral Therapy to Treat Insomnia: A Systematic Review and Meta-Analysis. PLoS One.

[51] Bureau of Labor Statistics. Workers on Flexible and Shift Schedules in May 2004. http://www.bls.gov/news.release/pdf/flex.pdf. (2005).

