# Author Correction: Validation of the Sleep Regularity Index in Older Adults and Associations with Cardiometabolic Risk

**DOI:** 10.1038/s41598-020-59762-1

**Published:** 2020-02-14

**Authors:** Jessica R. Lunsford-Avery, Matthew M. Engelhard, Ann Marie Navar, Scott H. Kollins

**Affiliations:** 1 Department of Psychiatry and Behavioral Sciences, Duke University Medical Center, Durham, N.C USA; 2 Duke Clinical Research Institute, Durham, NC USA

Correction to: *Scientific Reports* 10.1038/s41598-018-32402-5, published online 21 September 2018

The Article contains an error in Equation 3.

$$\frac{1440}{2\pi }\arctan 2(\mathop{\sum }\limits_{j=1}^{M}\mathop{\sum }\limits_{i=1}^{M}{s}_{i,j}\,\sin \,\frac{2\pi {t}_{i}}{1440}+\mathop{\sum }\limits_{j=1}^{M}\mathop{\sum }\limits_{i=1}^{M}{s}_{i,j}\,\cos \,\frac{2\pi {t}_{i}}{1440})$$

should read:

$$\frac{1440}{2\pi }\arctan 2(\mathop{\sum }\limits_{j=1}^{M}\mathop{\sum }\limits_{i=1}^{M}{s}_{i,j}\,\sin \,\frac{2\pi {t}_{i}}{1440},\mathop{\sum }\limits_{j=1}^{M}\mathop{\sum }\limits_{i=1}^{M}{s}_{i,j}\,\cos \,\frac{2\pi {t}_{i}}{1440})$$

Additionally, the Article contains a typographical error in the Methods section under subheading ‘Calculation of Sleep Regularity Index (SRI) and other sleep indices’ where,

“Sleep midpoint, our index of sleep timing, was calculated as a mean of circular quantities (appropriate for time of day) using the following equation (3), where ${t}_{j}$ denotes time of day in minutes at epoch ${j}$:”

should read:

“Sleep midpoint, our index of sleep timing, was calculated as a mean of circular quantities (appropriate for time of day) using the following equation (3), where ${t}_{j}$ denotes time of day in minutes at epoch $j$, and ${\rm{\arctan }}2(x,{y})$ denotes the angle between $(x,{y})$ and the positive $x$-axis.”

