# Author Correction: Validation of the Sleep Regularity Index in Older Adults and Associations with Cardiometabolic Risk

**DOI:** 10.1038/s41598-021-03253-4

**Published:** 2021-12-16

**Authors:** Jessica R. Lunsford-Avery, Matthew M. Engelhard, Ann Marie Navar, Scott H. Kollins

**Affiliations:** 1 Department of Psychiatry and Behavioral Sciences, Duke University Medical Center, Durham, NC USA; 2 Duke Clinical Research Institute, Durham, NC USA

Correction to: *Scientific Reports* 10.1038/s41598-018-32402-5, published online 21 September 2018

The original version of this Article contained errors in Table 2, where the “Irregular” and “Regular” “Median (IQR)” for “Total Sleep Time (minutes)” was incorrectly stated in 30-second epochs instead of minutes. Furthermore, the population medians for “Sleep in Clock Day (minutes)”, “Sleep in Clock Night (minutes)” and “Daily Light Exposure (minutes > 250 lux)” contained errors. The first 30-second epoch of each hour was not included when counting sleep and light exposure in the clock day and night.

The original Table 2 and accompanying legend appear below.

**Table 2:** Group differences in sleep-related variables among irregular and regular sleepers.

	Irregular	Regular	p-value	Cohen’s d
	Median (IQR)			
Actigraphy Measures				
Total Sleep Time (minutes)	954.3 (284.1)	961.2 (149.2)	0.604	0.089
Sleep Midpoint (clock time)	03:36 am (2:17)	02:59 am (1:14)	< 10^−9^	0.426
Physical Activity (counts in thousands)	184.3 (108.8)	229.3 (97.1)	< 10^−13^	0.517
Sleep in Clock Day (minutes)	76.0 (79.2)	8.9 (18.9)	< 10^−20^	1.543
Sleep in Clock Night (minutes)	410.7 (123.2)	469.8 (66.2)	< 10^−20^	0.889
Daily Light Exposure (minutes > 250 lux)	85.2 (108.3)	133.4 (140.8)	< 10^−11^	0.489
Self-Report Measures				
ESS	6 (6)	4 (5)	< 10^−15^	0.595
MEQ	17 (5)	18 (5)	< 10^−4^	0.315

As a result, the minute values in the Results’ sub-section “Group differences in sleep timing and light exposure” have been corrected.

“Specifically, on average, irregular sleepers slept for 65 minutes more during the clock day and 59 minutes less during the clock night compared to regular sleepers. In addition, irregular sleepers tend to be exposed to fewer minutes of light (>250 lux). On average, irregular sleepers were exposed to 48.2 fewer minutes of light per day than regular sleepers.”

now reads:

“Specifically, on average, irregular sleepers slept for 66.5 minutes more during the clock day and 60.4 minutes less during the clock night compared to regular sleepers. In addition, irregular sleepers tend to be exposed to fewer minutes of light (>250 lux). On average, irregular sleepers were exposed to 48.4 fewer minutes of light per day than regular sleepers.”

The original Article has been corrected.

