# Supplementary material for: Validation of the Sleep Regularity Index in Older Adults and Associations with Cardiometabolic Risk
Source: Sci Rep. 2018 Sep 21;8:14158. doi: 10.1038/s41598-018-32402-5 (PMC6154967; doi:10.1038/s41598-018-32402-5)
Supplement: Supplementary file 1 — Supplementary Information [file 41598_2018_32402_MOESM1_ESM.docx]

**Supplementary Information**

Title: Validation of the Sleep Regularity Index in Older Adults and Associations with Cardiometabolic Risk

Authors: Jessica R. Lunsford-Avery, Matthew M. Engelhard, Ann Marie Navar, Scott H. Kollins


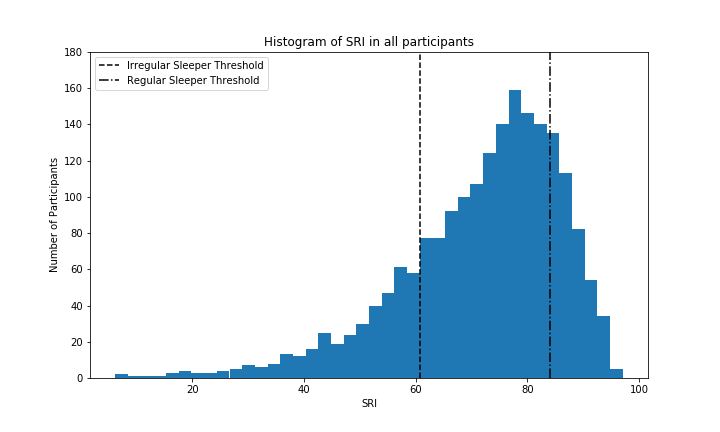


**Supplementary Figure S1**. Distribution of SRI in the MESA sample.


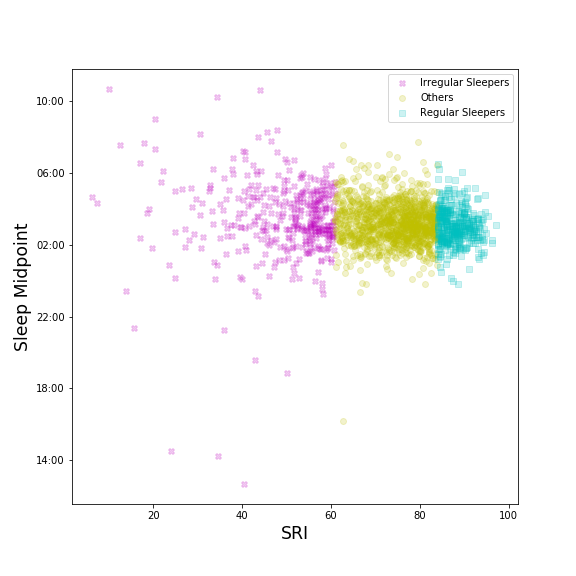


**Supplementary Figure S2**. Relationship between SRI and Sleep Midpoint


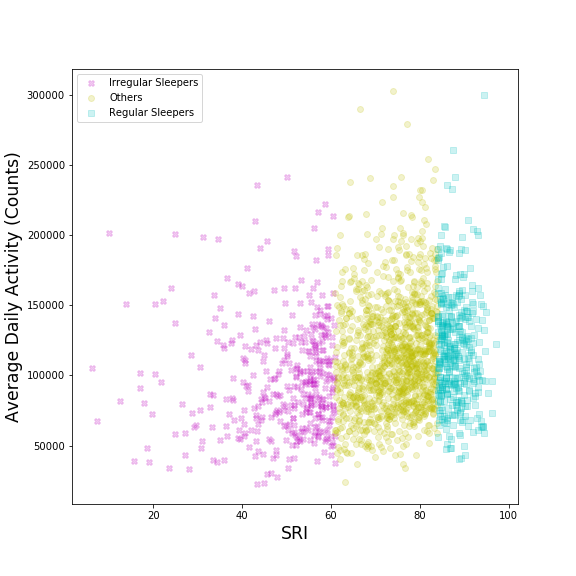


**Supplementary Figure S3**. Relationship between SRI and Physical Activity
